# A Novel Iminosugar UV-12 with Activity against the Diverse Viruses Influenza and Dengue (Novel Iminosugar Antiviral for Influenza and Dengue)

**DOI:** 10.3390/v7052404

**Published:** 2015-05-13

**Authors:** Kelly L. Warfield, Emily Plummer, Dominic S. Alonzi, Gary W. Wolfe, Aruna Sampath, Tam Nguyen, Terry D. Butters, Sven G. Enterlein, Eric J. Stavale, Sujan Shresta, Urban Ramstedt

**Affiliations:** 1 Unither Virology LLC, Silver Spring, MD 20910, USA; E-Mails: asampath@unithervirology.com (A.S.); urban_ramstedt@yahoo.com (U.R.); 2 La Jolla Institute for Allergy and Immunology, La Jolla, CA 92037, USA; E-Mails: eplummer@liai.org (E.P.); sujan@lji.org (S.S.); 3 Oxford Glycobiology Institute, Oxford OX1 3QU, UK, E-Mails: dominic.alonzi@bioch.ox.ac.uk (D.S.A.); terry.butters@btinternet.com (T.D.B.); 4 Gary Wolfe Toxicology, Herndon, VA 20170, USA; E-Mail: gary@gwtox.com; 5 Tam Nguyen LLC, Gaithersburg, MD 20879, USA; E-Mail: tam.3.nguyen@gmail.com (T.N.); 6 Integrated Biotherapeutics, Gaithersburg, MD 20878, USA; E-Mails: sven@integratedbiotherapeutics.com (S.G.E.); eric@integratedbiotherapeutics.com (E.J.S.)

**Keywords:** glucosidase, glycosylation, *Flaviviridae*, flavivirus, *Orthomyxoviridae*, orthomyxovirus, mouse

## Abstract

Iminosugars are capable of targeting the life cycles of multiple viruses by blocking host endoplasmic reticulum α-glucosidase enzymes that are required for competent replication of a variety of enveloped, glycosylated viruses. Iminosugars as a class are approved for use in humans with diseases such as diabetes and Gaucher’s disease, providing evidence for safety of this class of compounds. The *in vitro* antiviral activity of iminosugars has been described in several publications with a subset of these demonstrating *in vivo* activity against flaviviruses, herpesviruses, retroviruses and filoviruses. Although there is compelling non-clinical *in vivo* evidence of antiviral efficacy, the efficacy of iminosugars as antivirals has yet to be demonstrated in humans. In the current study, we report a novel iminosugar, UV-12, which has efficacy against dengue and influenza in mouse models. UV-12 exhibits drug-like properties including oral bioavailability and good safety profile in mice and guinea pigs. UV-12 is an example of an iminosugar with activity against multiple virus families that should be investigated in further safety and efficacy studies and demonstrates potential value of this drug class as antiviral therapeutics.

## 1. Introduction

Iminosugars that are glucomimetics function as competitive endoplasmic reticulum (ER) α-glucosidase inhibitors and have antiviral activity through a host based mechanism. For these reasons, iminosugars offer an attractive avenue for developing broad spectrum antiviral therapies [1,2]. Viruses that require use of the host-cell glycosylation pathway for replication are susceptible to targeting by the iminosugar class of drugs. Blocking this glycosylation pathway via inhibition of the α‑glucosidase enzymes in the ER leads to the misfolding of viral glycoproteins that are subsequently sent to the proteasome for degradation and elimination or to the production of defective daughter virions comprised of misfolded proteins [2,3,4,5]. Key data validating ER α-glucosidases as an antiviral target are provided by a recent publication by Sadat *et al.* [6]. The authors identified two siblings with a spectrum of developmental abnormalities, but with no history of viral disease in spite of significant hypogammaglobulinemia. The underlying genetic defect is knock-out of the ER α-glucosidase I, a target enzyme for our inhibitor program. Neither of the siblings were able to generate appropriate immune responses to live viral vaccines including measles, mumps, rubella, and varicella and cells from these subjects are deficient in uptake and maturation of multiple divergent viruses including HIV and influenza. This report supports that pharmacological inhibition of the ER α‑glucosidases should result in broad-spectrum antiviral effects.

Our host-based, broad-spectrum antiviral drug platform is based on iminosugar analogs of N-butyl-deoxynojirimycin (NB-DNJ or miglustat), which is approved for use in humans. NB-DNJ is an orally available, relatively inexpensive to manufacture drug that is safe and is used for treatment of Gaucher’s disease [7]. NB-DNJ has also been shown to exhibit broad-spectrum antiviral activity *in vitro* against viruses including DENV, HCV, and HIV but requires concentrations (>30 μM) that are unreasonable to achieve *in vivo* [3]. Another well described iminosugar, castanospermine, demonstrates more potent antiviral activities against a range of viruses including flaviviruses, herpesviruses, influenza virus (INFV) and retroviruses [8,9,10,11,12]. Additional validation of this approach is provided in recent publications describing iminosugar ER α-glucosidase inhibitors with efficacy against diverse viruses including flaviviruses, influenza virus and filoviruses in mice [13,14,15,16,17,18].

**Figure 1:**
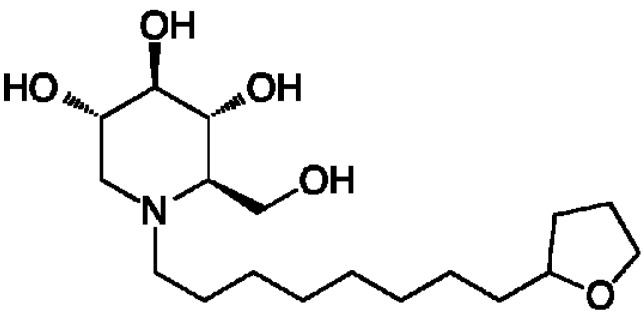
Structure of the iminosugar UV-12.

By targeting a set of host enzymes, we expect to overcome liabilities of directly acting antivirals. Using iminosugars to target the host ER α-glucosidases that are critical for replication of a wide variety of viral families having properties of glycosylated structural proteins and enveloped virions, it is expected that a single drug could be used for multiple acute viral infections. Use of a host-targeted antiviral is not expected to result in selection of drug-resistant viral strains since pressure is not directly exerted on the virus itself during replication [19]. Here we describe a novel iminosugar (2R,3R,4R,5S)-2-(hydroxymethyl)-1-(8-(tetrahydrofuran)-2-yl)octyl)piperidine-3,4,5-triol that we named UV-12 (structure shown in Figure 1), having strong drug-like properties and *in vivo* activity against the divergent dengue (DENV) and influenza viruses.

## 2. Materials and Methods

### 2.1. Inhibition of α-Glucosidases

#### 2.1.1. Purified Enzyme Inhibition

The assays for ER α-glucosidases I and II [20] used enzymes purified from rat liver as previously described [21,22]. Oligosaccharide substrates Glc_(1-3)_Man_(4-7)_GlcNAc_(1-2)_ were isolated from cultured cells treated with an α-glucosidase inhibitor, NB-DNJ, and purified by normal phase high-performance liquid chromatography (NP-HPLC) following fluorescent labeling [20]. Enzyme was incubated for up to 2 h with oligosaccharide substrate and UV-12 at various concentrations. The reaction was terminated and the products separated by NP-HPLC. The amount of digestion was measured in comparison to control (no inhibitor) and dose-response data plotted using a four-parameter logistic model (Hill-Slope). The 50% inhibitory concentration (IC_50_) value was calculated for α-glucosidase I (Glc3 substrate) and α-glucosidase II (Glc2 substrate and Glc1 substrate).

#### 2.1.2. Cellular Inhibition of Endoplasmic Reticulum α-Glucosidase Activity Using Free Oligosaccharide (FOS) Assay

We have previously reported a cell-based assay for evaluation of effects of iminosugars on ER α‑glucosidases [20]. Briefly, human (HL60) cells were cultured to a high density (1 × 10^7^ cells/mL) in the presence of UV-12 in fresh medium containing at multiple concentrations for 24 h before cells were harvested, washed and extracted for free oligosaccharides (FOS). The cells were seeded at a lower density to achieve a high density at the end of the incubation period. Following cell culture, the medium was removed and the cells were washed three times with PBS by centrifugation. Washed cells were stored at −20 °C for a short time before thawing and Dounce homogenization in water. The maximum recovery of FOS was performed using the following conditions. The homogenate from 0.1–0.2 mg protein was desalted and deproteinated by passage through a mixed-bed ion-exchange column [0.2 mL of AG50W-X12 (H^+^, 100–200 mesh) over 0.4 mL of AG3-X4 (OH^−^, 100–200 mesh)], pre-equilibrated with water (5 × 1 mL). The homogenate was added to the column which was washed with 4 × 1 mL of water, and the eluate collected. The extracted and purified FOS were then dried under vacuum or by lyophilizing. The FOS were labelled with 2-AA and purified using DPA-6S columns as described previously [20]. Labelled oligosaccharides in 50 mM Tris/HCl buffer, pH 7.2, were purified using a ConA (Concanavalin A)-Sepharose 4B column (100 μL packed resin). The column was pre-equilibrated with 2Å~1 mL of water followed by sequential 1 mL washes of 1 mM MgCl_2_, 1 mM CaCl_2_, 1 mM MnCl_2_ and 2Å~1 mL of 50 mM Tris/HCl buffer, pH 7.2. The sample was added and allowed to pass through the column before washing with 2Å~1 mL of 50 mM Tris/HCl buffer, pH 7.2. The ConA-bound FOS were then eluted with 2 × 1 mL of hot (70 °C) 0.5 M methyl α-d-mannopyranoside in 50 mM Tris/HCl buffer, pH 7.2. The purified and fluorescently labelled FOS were separated by NP-HPLC [20].

Free glucosylated oligosaccharides were measured as a biochemical product of ER α-glucosidase I and II inhibition. The peak areas of glucosylated FOS corresponding to Glc_1_Man_4_GlcNAc_1_ and Glc_3_Man_5_GlcNAc_1_, and in some instances Glc_3_Man_7_GlcNAc_2_, were measured and used to determine the relative amount of α-glucosidase I and II inhibition in cells.

#### 2.1.3. Cellular Half-Life of Endoplasmic Reticulum α-glucosidase I Blockage

MDBK cells were treated with 100 μM UV-12 for 24 h to generate Glc_3_Man_7_GlcNAc_2_ [23]. UV12 was removed and cells cultured for 96 h. The level of Glc_3_Man_7_GlcNAc_2_ was monitored using the FOS assay (Section 2.1.2) every 24 h. Half-life was determined as the time when the level of Glc_3_Man_7_GlcNAc_2_ declined to 50% of the initial accumulation.

### 2.2. Cytotoxicity and in Vitro Antiviral Activity

#### 2.2.1. Cytotoxicity

The cytotoxicity of UV-12 in Madin-Darby Canine Kidney (MDCK) and Vero cells was determined based on the manufacturer’s instructions using a commercially available kit (CellTiter-Glo Luminescent Cell Viability Assay (Promega, Madison, WI, USA)). UV-12 was tested at 6 concentrations starting at 500 μM with subsequent two-fold dilutions. Cytotoxicity was measured after 5 days. The 50% cellular cytotoxicity (CC_50_) value was determined by comparing the compound-treated samples with vehicle-only treated cells set as 100% survival and no cells set as 0% survival.

#### 2.2.2. *In Vitro* Antiviral Activity

The *in vitro* antiviral activity of UV-12 was tested using a virus yield-plaque assay or tissue culture infectious dose (TCID) format to determine the reduction in titer of virus after growth in the presence of multiple concentrations of compound. Viruses used for these studies included DENV (serotype 2, New Guinea C isolate propagated in Vero cells), INFV (mouse-adapted A/Texas/36/91 (H1N1) propagated in MDCK cells) or Venezuelan Equine Encephalitis virus (VEEV, TC-83 vaccine strain propagated in Vero cells). UV-12 was tested at 8 concentrations starting at 250 μM with subsequent two-fold dilutions in Vero (DENV and VEEV) or MDCK (INFV) cells. UV-12 was added to cells 1 h before infection, DENV, INFV or VEEV was used for infection at an MOI of 0.01, 0.01 or 0.1, respectively. Supernatants were harvested and clarified of cell debris after 5 days for DENV and INFV or 3 days for VEEV and analyzed for virus content using an immunoplaque (DENV), TCID (INFV) or plaque (VEEV) assay. A four-parameter logistic curve was used to generate 50% inhibitory concentration (IC_50_) was calculated using XLFit equation 205 based on percent reduction of virus yield compared to the virus titer in media-treated cells.

### 2.3. In Vitro Absorption-Distribution-Metabolism-Elimination (ADME) Studies

UV-12 was tested for drug-likeness using a panel of standard assays including ADME profiling for plasma protein binding, metabolic stability in human liver microsomes, permeability/efflux, solubility, Cytochrome P450 (CYP) inhibition, and hERG binding. These assays were performed by Advinus Therapeutics Limited (Bangalore, India).

#### 2.3.1. Protein Binding Studies in Mouse, Rat, Dog and Human Plasma

Protein binding of UV-12 was determined in mouse, rat, dog and human plasma. Each plasma sample was spiked with a single concentration (10 μM) of compound, incubated for 6 h, dialyzed against buffer and liquid chromatography tandem mass spectrometry (LC-MS/MS) was used to determine the unbound and bound percentages of UV-12 to the plasma proteins.

#### 2.3.2. Metabolic Stability in Mouse, Rat, Dog and Human Liver Microsomes

The *in vitro* metabolic stability of UV-12 in mouse, rat, dog and liver microsomes was evaluated at one substrate concentration (0.5 µM), one protein concentration (0.5 mg/mL), and ten time points in duplicate (0, 3, 6, 9, 12, 15, 18, 21, 27 and 30 min). LC-MS/MS was used to monitor the clearance of each compound to calculate the half-life (T½).

#### 2.3.3. Permeability/Efflux Ratio in Caco-2 Cells

The *in vitro* apparent permeability of UV-12 at 10 µM was determined across a Caco-2 cell monolayer to assess intestinal transport in both directions (apical to basolateral, A:B, and basolateral to apical, B:A). LC-MS/MS was used to determine the UV-12 concentration.

#### 2.3.4. Solubility

Aqueous solubility of the test compounds was be tested in phosphate buffered saline (PBS) at 10, 20, 40, 60, 80 and 100 μM with LC-MS/MS as the detection method.

#### 2.3.5. Inhibition of Cytochrome P450 1A2, 2C9, 2C19, 2D6 and 3A4 Isoenzymes

The *in vitro* inhibition of cytochrome P450 (CYP) 1A2, 2C9, 2C19, 2D6 and 3A4 isozymes by UV-12 was evaluated in human liver microsomes by monitoring production of selected metabolites following incubation with probe substrates using LC-MS/MS detection. For each isozyme, a standard CYP-specific probe substrate was incubated along with human liver microsomes and cofactors, and production of selected metabolite was measured. The inhibitory effect of increasing concentrations of UV-12 up to 100 μM on the production of the metabolite was determined, and the concentration of inhibitor required for a 50% reduction in the measured enzyme activity was estimated.

#### 2.3.6. Functional hERG Assay

To test the ability of UV-12 for potential to inhibit the hERG (human ether-a-go-go-related gene) channel, three concentrations (30, 100 and 300 μM) were tested using a standard automated whole-cell patch clamp method using a Chinese Hamster Ovary (CHO) cell line stably transfected with hERG gene [24].

#### 2.3.7. Ames Test

UV-12 was tested for mutagenic potential in the miniaturized version of the Ames (mini-Ames) assay using histidine auxotrophic strains of *Salmonella typhimurium* TA98, TA100 and TA1535 [25]. The bacterial tester strains were exposed to the test item in the presence and absence of metabolic activation system (S-9 fraction prepared from Aroclor 1254 induced rat liver). UV-12 was tested at doses of 1.5, 5, 15, 50, 150, 500, 1500 and 5000 μg per plate along with DMSO as vehicle control and appropriate positive controls in a direct plate incorporation assay.

### 2.4. In Vivo Efficacy Studies

#### 2.4.1. UV-12 Preparation and Treatment Regimens

Mice were treated orally three times daily (TID at eight hour intervals) with UV-12 dissolved in acidified water at 20–100 mg/kg/dose for 7 (DENV) or 10 days (INFV) starting at −1 h relative to infection unless otherwise indicated. UV-12 was delivered in 50 (DENV) or 100 (INFV) μL per dose. Water-only was used as the negative control treatment in all studies delivered with the same regimen as UV-12.

#### 2.4.2. Health Assessments, Early Endpoints and Oversight

Weights, health scores and temperatures were monitored and recorded daily for the duration of the study on individual mice. A standard health scoring system from 1–7 was utilized where scores indicated the following: 1, healthy; 2, slightly ruffled; 3, ruffled; 4, sick; 5, very sick; 6, moribund; and 7, found dead. Mice were sacrificed at a health score greater than or equal to 5 or when a weight loss of >30% of their original weight was recorded. Animals were euthanized in accordance with the 2013 American Veterinary Medical Association (AVMA) Guidelines on Euthanasia using carbon dioxide exposure followed by cervical dislocation. All experimental procedures and studies were preapproved and performed according to guidelines set by the Noble Life Sciences Animal Care and Use Committee for influenza virus studies (protocol 10-09-003-IBT) and the La Jolla Institute for Allergy and Immunology Animal Care and Use Committee for studies with dengue virus (protocol AP028-SS1-0612).

#### 2.4.3. Influenza Efficacy Studies

Influenza efficacy studies were performed as previously described [18]. Groups of ten 6–8 week-old female BALB/c mice (Charles River Labs) were microchipped (Bio Medic Data Systems) for identification and temperature monitoring at least 3 days prior to infection. Mice were infected with approximately 52 plaque-forming units (PFU) of A/Texas/36/91 (H1N1) diluted in phosphate-buffered saline (PBS) via intranasal administration under light anesthesia with isoflurane. Mice were treated via oral gavage with UV-12 at indicated time relative to infection at various concentrations three times daily. After challenge, mice were monitored at least daily for weights, health and survival for a total of 14 days.

#### 2.4.4. Dengue Efficacy Studies

The animal model used for this study is AG129 (129/Sv IFN-α/β and -γ receptor deficient) mice infected with 1 × 10^4^ pfu via IV injection with Dengue 2 (DENV2) strain S221 with antibody dependent enhancement (ADE), which die of TNF-α mediated acute/early death by day 4–5. AG129 mice of both sexes and aged 5–6 weeks at the start of the study were used as the test system for DENV. The AG129 mice were bred and housed under specific pathogen-free conditions at the La Jolla Institute (LJI). Mice were ear-tagged for identification. Generation and preparation of DENV2 strain S221 is described previously [26,27,28,29]. One hour before virus infection, 5 μg of the monoclonal antibody 2H2 (anti-prM/M) was administered in 200 μL PBS via intraperitoneal injection (IP) to induce the antibody dependent enhancement-mediated DHF/DSS-like disease. The challenge virus, DENV2 S221 was administered in a volume of 200 μL (injected virus diluted in PBS + 5% FCS) via intravenous (IV) tail vein injection with 10^9^ GE (genomic equivalents).

To assess the efficacy of UV-12 against a lethal DENV2 infection, AG129 mice were treated with 20 or 100 mg/kg of UV-12 orally thrice daily for 7 days starting 1 h before challenge using the ADE DENV model. Mice were monitored at least daily for health, weight and survival for 10 days total. To assess the efficacy of UV-12 in reducing the viral load and modifying the cytokine responses of mice challenged with dengue virus, UV-12 was administered orally starting at 1 h before infection and delivered three times daily until the time of sampling at 72 or 96 h post infection. Viral titers in the serum or liver, spleen, kidney and small intestines were determined by qRT-PCR for each sample from individual animals and the mean with standard deviation are also shown for each group. Serum was separated using serum collection tubes and viral RNA was isolated from serum using Qiagen Viral RNA isolation kits. Brains were removed and immediately stored in RNAlater at 4 °C. Organs were homogenized and total RNA was isolated using Qiagen RNeasy isolation kits. Dengue virus 2 qRT-PCR was performed on all samples, and genomic equivalent (GE) per ml of serum or in tissue was determined as previously described [28]. Data are shown as log DENV genome equivalents per mL of serum or as log DENV genome equivalents per relative 18S (×10^4^) for the tissues analyzed. Serum from control (water) and UV-12 treated animals were also tested for levels of eotaxin, G-CSF, GM-CSF, IFN-γ, IL-1α, IL-1β, IL-2, IL-3, IL-4, IL-5, IL-6, IL-9, IL-10, IL-12 (p40), IL-12 (p70), IL-13, IL-17A, KC, MCP-1 (MCAF), MIP-1α, MIP-1β, RANTES and TNF-α (Bio-Plex Pro™ Mouse Cytokine 23-plex Assay). All samples were analyzed as recommended by the manufacturer (Bio-Rad, Hercules, CA, USA).

#### 2.4.5. Statistical Analysis

Survival data was analyzed in GraphPad Prism using log-rank analysis. Cytokine data and viral titer data were analyzed using GraphPad Prism using a two-tailed *t*-test.

### 2.5. In Vivo Pharmacokinetic (PK) and Safety Studies

#### 2.5.1. Sample Analysis

A fit-for-purpose LC-MS/MS method with a lower limit of quantification of 5.10 or 10.01 ng/mL for mice or guinea pigs, respectively, was used for quantification of UV-12 in plasma samples. The pharmacokinetic parameters of UV-12 were calculated using the non-compartmental analysis tool of WinNonlin^®^ software (Version 5.2, Pharsight Corporation, St. Louis, MO, USA).

#### 2.5.2. Mouse PK Analysis

To investigate the bioavailability and PK of UV-12, male Swiss Albino mice (*n* = 24 total) were administered UV-12 via IV injection or oral gavage. Blood samples (*n* = 3 per time point) were collected at 0.083 (only IV), 0.25, 0.5, 1, 2, 4, 8 and 16 h post-dose. At each collection time, approximately 120 µL of blood was withdrawn from retro orbital plexus and transferred to pre-labeled microfuge tube containing 200 mM K_2_-EDTA solution (20 µL per mL of blood) as anticoagulant. Blood samples were centrifuged at 5000 g for 5 min at 4 ± 2 °C to separate plasma and stored below −60 °C until bioanalysis. These studies were performed at Advinus Therapeutics Limited (Bangalore, India) under approved study numbers N1883 and N1884).

#### 2.5.3. Guinea Pig Pharmacokinetics and Maximum Tolerated Dose

To determine the maximum tolerated dose of UV-12 in female Hartley guinea pigs, doses of 20, 40, 60, 80 and 100 mg/kg body weight was administered by the intramuscular route and at the doses of 10, 20, 40, 60 and 80 mg/kg body weight by the IV route. The oral route was not selected for guinea pig dosing due to difficulties with repeated oral gavage procedures in this species. The different concentrations of UV-12 were administered at an equivolume dose of 2 mL/kg bodyweight. The animals (*n* = 3/group) were observed for clinical signs and mortality and gross necropsy was performed on the dead animals and the animals surviving the observation period of 7 days.

To assess the bioavailability and pharmacokinetic parameters of UV-12 in Hartley guinea pigs, animals were administered a single subcutaneous, intramuscular or IV bolus of 100, 100 or 25 mg/kg body weight of UV-12, respectively. A total of 9 female guinea pigs were used in this study. Blood samples from each guinea pig were collected at pre-dose and at 0.083 (only for IV), 0.25, 0.5, 1, 2, 4, 8 and 16 h post-dose. At each time point, approximately 0.5 mL of blood was withdrawn from jugular vein of the cannulated guinea pig. The blood samples were collected into pre-chilled (at 4 °C) labeled tubes containing 200 mM K_2_-EDTA solution (20 μL per mL of blood). Following sampling equal volume of heparinized saline was flushed into the catheter. The blood samples were centrifuged at 5000 g for 5 min at 4 ± 2 °C within 30 min of scheduled time. The plasma samples were placed in labeled tubes and immediately stored below −60 until their bioanalysis.

These studies were performed at Advinus Therapeutics Limited under approved study numbers N1819-N1822.

#### 2.5.4. Repeat Dose Safety Study in Mice

To determine the toxicity of UV-12 following 10 day repeat dose oral (by gavage) administration to male Swiss Albino mice, a high dose level of 100 mg/kg body weight was selected. The vehicle control and treatment group consisted of 5 male mice each aged 8–9 weeks at the start of the study. The dose formulation was administered to each group three times a day with approximately 8 h between each administration for 10 consecutive days. The dosing volume administered to each mouse per administration was 0.3 mL/dose. All mice were observed for mortality and morbidity twice daily during treatment period. Parameters evaluated were mortality, clinical signs, body weights, food consumption, fasting body weight, clinical chemistry, organ weights, gross pathology and histopathology. The food consumption was measured during Days 1–4, 4–7 and 7–10 for all groups. The amount of spillage was considered for calculation of food consumption. All mice at the end of the treatment period (on Day 11) were fasted for 3 to 5 h (water allowed) and retro-orbital sinus was punctured to collect blood using a fine capillary tube under isoflurane anaesthesia. Blood samples were collected in tubes containing lithium heparinized tubes for determination of clinical chemistry parameters. Plasma was separated by centrifuging the whole blood samples at 4 °C, 5000 rpm for 10 min and analyzed using Roche/Hitachi 902 Analyzer (Hitachi High-Technologies Corporation, Tokyo, Japan) for the following parameters: Alanine Aminotransferase, Albumin, Alkaline phosphatase, Aspartate Aminotransferase, Blood Urea Nitrogen, Creatinine, Gamma Glutamyl Transpeptidase, Glucose, Inorganic phosphorous, Potassium, Sodium, and Total Cholesterol. All mice at the end of the treatment period were subjected to detailed necropsy and findings were recorded. All mice were fasted for 3 to 5 h (water allowed), weighed, anaesthetized with isoflurane, exsanguinated and subjected for gross examination. On completion of the gross pathology examination, a total of 43 tissues and organs were collected from each mouse. Selected organs and tissues were weighed. The organ weight ratios as percentage of body and brain weight was determined. 10% Neutral Buffered Formalin (NBF) was used for fixation. The tissues were processed for routine paraffin embedding and 4–5 micron sections were stained with Mayer’s Haematoxylin and Eosin stain. Histopathological examination was carried out on the preserved organs of all mice. Data for this study was captured using Provantis^TM^: Parameters such as body weight, net body weight gains (derived data), food consumption (derived data), terminal fasting body weight, laboratory investigations-clinical chemistry, organ weights and their ratios data (derived data) was analyzed using Provantis^TM^ built-in statistical tests. All analyses and comparisons were evaluated at the 5% (*p* ≤ 0.05) level. This study was performed at Advinus Therapeutics Limited under approved study number N1896).

## 3. Results

### 3.1. In Vitro α-Glucosidase Activity of UV-12

UV-12 inhibits both α-glucosidase I and II in a purified enzyme assay with IC_50_ ranging from 0.14 µM for α-glucosidase I to 0.83–1.1 µM for α-glucosidase II (Table 1). The assay uses purified α-glucosidases I and II from rat liver and shows UV-12 to have a greater inhibition against α-glucosidase I compared to α-glucosidase II. There was no significant difference in the inhibitory potential of UV-12 against the two activities of α-glucosidase II *in vitro*.

**Figure 2:**
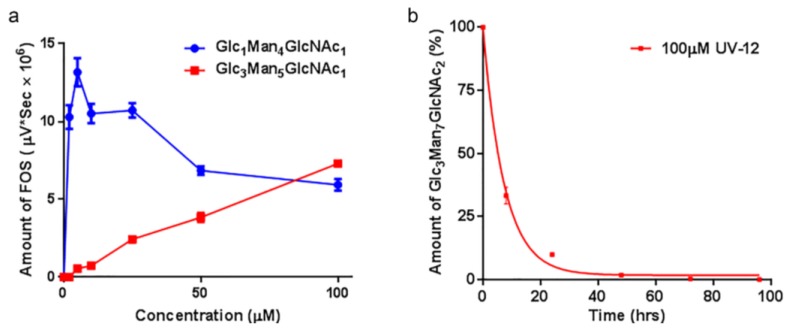
Rates of glucosylated FOS generated in the presence of the α-glucosidase inhibitor UV-12. (**a**) Levels of a monoglucosylated and triglucosylated FOS species generated over a concentration range of UV-12 are shown. The products of glucosidase II inhibition (Glc_1_Man_4_GlcNAc_1_ as the exemplar) and glucosidase I inhibition (Glc_3_Man_5_GlcNAc_1_ as the exemplar) are indicated; (**b**) Cellular half-life of UV-12 determined based on ER α-glucosidase I inhibition (generation of Glc_3_Man_7_GlcNAc_2_ as the readout) are indicated. Fluorescently labeled FOS species were isolated from cells at various times following treatment with 100 μM UV-12. The mean and SD from triplicate assays is shown in each graph.

UV-12 also inhibited ER α-glucosidase enzymes in a cell-based assay (Figure 2a). The FOS assay uses glycan biomarkers of ERAD to monitor α-glucosidase I and II inhibition. The assay shows UV-12 causes an initial increase in the levels of mono-glucosylated free glycans at low concentrations, and therefore α-glucosidase II inhibition. Subsequently, there is an increase in tri-glucosylated FOS as UV-12 concentrations are increased, allowing demonstration of ER α-glucosidase I inhibition. Since α-glucosidase I functions at an earlier step in the N-linked glycoprotein biosynthetic pathway, a decrease in the level of mono-glucosylated free glycans produced in the cell is not observed until higher UV-12 concentrations.

The cellular half-life of UV-12 inhibition as shown by blockage of α-glucosidase I is 5.11 ± 1.82 h in MDBK cells (Figure 2b) when monitoring the biomarker of this inhibition in the ER [23]. Glc3Man7GlcNAc2 is produced in the ER upon α-glucosidase I inhibition by UV-12 at 50 µM and the level of this FOS species can be followed upon removal of UV-12. This demonstrates that the α-glucosidase blockage has been removed with the removal of the terminal glucose from the Glc3Man7GlcNAc2 FOS species in the ER occurring by the activity of α-glucosidase I and demonstrates that UV-12 is a reversible inhibitor in cells.

**Table 1:** Iminosugars are potent ER α-glucosidase inhibitors and can inhibit viral replication *in vitro*. UV-12 was tested for the ability to inhibit rat liver ER α-glucosidases I and II on oligosaccharide substrates, Glc_(1-3)_Man_(4-7)_GlcNAc_(1-2)_ isolated from cultured cells. The mean and SD of the IC_50_ from triplicate assays is shown. Viral inhibition was tested using a yield/plaque assay with multiple concentrations of UV-12. The mean from duplicate assays is shown.

Compound	α-glc I	α-glc II		Virus		
	Glc_3_	Glc_2_ Glc_1_		DENV-2	INFV	VEEV
UV-12	0.14 ± 0.10 μM	1.10 ± 0.48 μM	0.83 ± 0.37 μM	21.71 μM	>250 μM	69.4 μM

### 3.2. Antiviral Activity of UV-12

#### 3.2.1. *In Vitro* Activity of UV-12

The CC_50_ of UV-12 in Vero and MDCK cells was >500 μM. To assess the number and infectivity of daughter virions produced in the presence of UV-12, a yield assay followed by plaque assay (DENV and VEEV) or TCID (INFV) was used. UV-12 inhibited DENV-2 and VEEV but not INFV *in vitro* (Table 1). The IC_50_ for UV-12 was 21.71 µM for DENV-2, 69.4 µM for VEEV, and >250 µM for INFV (H1N1).

#### 3.2.2. Antiviral Activity of UV-12 against Influenza

**Figure 3:**
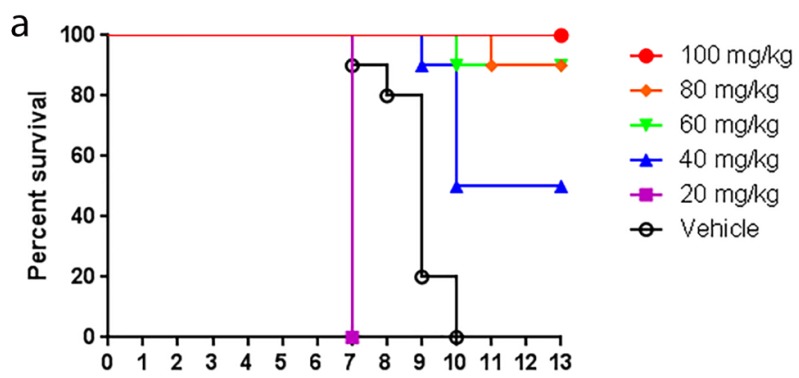

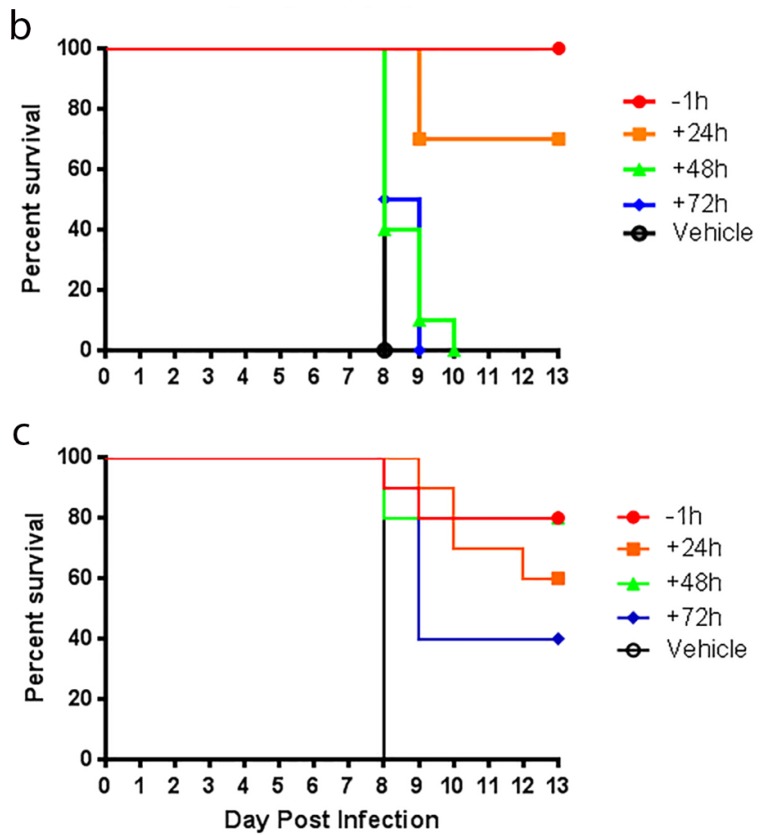
Protection by UV-12 in an INFV H1N1 mouse model. (**a**) BALB/c mice (*n* = 10/group) were treated orally with 100, 80, 60, 40 or 20 mg/kg of UV-12 or vehicle only thrice daily for 10 days starting 1 h before infection with ~1 LD_90_ of mouse-adapted INFV A/Texas/36/91 (~52 plaque forming units); (**b**)–(**c**) BALB/c mice (*n* = 10/group) received the first treatment dose of 100 mg/kg (**b**) or 60 mg/kg (**c**) of UV-12 starting at −1, 24, 48, or 72 h relative to infection with ~1 LD_90_ of INFV A/Texas/36/91 via IN instillation. Treatment began at the time point indicated and continued TID every 8 h for a total of 10 days.

We have recently demonstrated efficacy of the UV-4 iminosugar against influenza [18]. In order to determine whether UV-12 could also promote survival of mice infected with INFV, UV-12 was delivered ~60 min prior to viral challenge with lethal mouse-adapted INFV A/Texas/36/91 (H1N1) via oral gavage at 100, 80, 60, 40, or 20 mg/kg and continued three times daily for 10 days. While UV-12 did not inhibit *in vitro* INFV replication, treatment with UV-12 dosed thrice daily for 10 days protected mice challenged with INFV (Figure 3a). Mice that were treated with 100 mg/kg of UV-12 displayed 100% survival and the groups that were treated with 80 and 60 mg/kg each displayed 90% survival. The group treated with 40 mg/kg of UV-12 displayed 50% survival and a mean survival time of 9 days, while the vehicle-control group and group treated with 20 mg/kg displayed 0% survival and a mean survival of 9 and 7 days, respectively. Therefore, the minimum effective dose for UV-12 is 60 mg/kg (Figure 3a).

To determine the therapeutic window of UV-12 in the influenza mouse model, UV-12 was administered starting at −1, +24, +48, +72 h relative to challenge at 100 (Figure 3b) or 60 mg/kg (Figure 3c). At the dose of 100 mg/kg, mice that were treated starting at −1 h displayed 100% survival and the group that was treated at +24 h displayed 70% survival. Mice that were treated starting at +48 or +72 h post-infection displayed 0% survival. In these groups, 100% mortality was delayed by one day compared to the vehicle (water) control. When UV-12 was administered at 60 mg/kg, the groups that were treated at −1, +24 h, +48, and +72 h displayed 80, 60, 80, and 40% survival, respectively, compared to mice treated with vehicle in the study displaying 0% survival. Therefore, UV-12 dosed at 100 or 60 mg/kg can provide protection when dosing starts after infection.

#### 3.2.3. Antiviral Activity of UV-12 against Dengue

**Figure 4:**
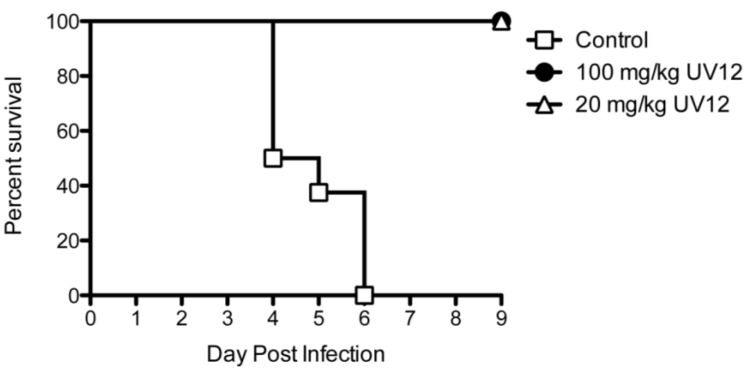
Protection by UV-12 in DENV ADE mouse model. AG129 mice were treated with 100 or 20 mg/kg of UV-12 orally thrice daily for 7 days starting 1 h before challenge using the ADE DENV model.

*In vivo* efficacy of UV-12 was also tested using a DENV antibody-dependent enhancement (ADE) in AG129 mice lacking both type I and type II interferon receptors. In this model, the virus (1 × 10^4^ pfu) is administered in combination with 15 µg of monoclonal antibody 2H2 (ATCC, Manassas, VA, USA) one hour before the viral challenge [16,27]. Using this ADE AG129 mouse model, UV-12 protected 100% of animals when administered at 100 or 20 mg/kg starting at 1 h before viral challenge (Figure 4). Mice treated with vehicle only had 0% survival and a mean time to death of 5 days.

**Figure 5:**
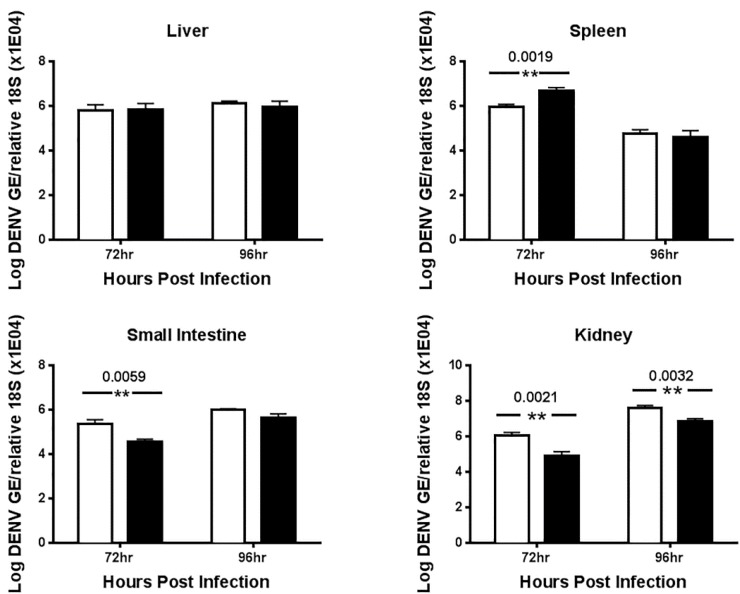

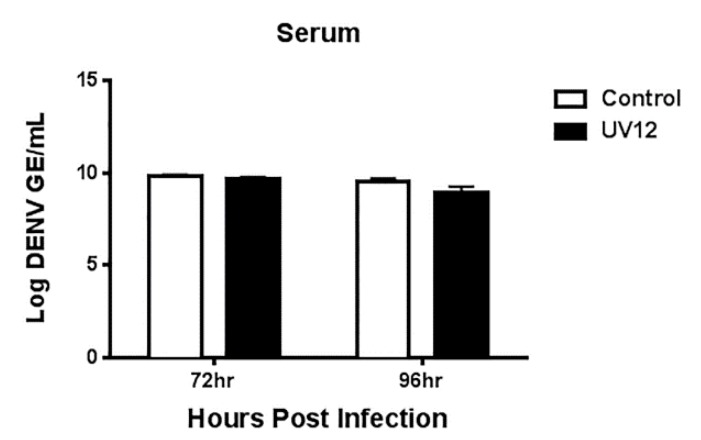
Dengue viral titer determined using qRT-PCR in serum or tissues collected from UV-12 treated AG129 mice. Groups of AG129 mice received the first treatment dose of 100 mg/kg of UV-12 or vehicle control 1 h before infection with DENV2 at a dose of ~1LD_90_. Treatment continued three times daily at 8 h intervals until tissue harvest at 72 or 92 h post infection. Viral titers in the serum or liver, spleen, kidney and small intestines were determined by qRT-PCR for each sample from individual animals and the mean with standard deviation are also shown for each group. Data are shown as log DENV genome equivalents per mL of serum or as log DENV genome equivalents per relative 18S (× 10^4^) for the tissues analyzed. Statistical differences (*p* < 0.05) are indicated with the actual values over the relevant data points.

To examine the effect of UV-12 on virus replication on DENV *in vivo*, infected mice were treated with 100 mg/kg starting at 1 h before infection until the time of harvest. All the mice in this study were predetermined for sampling at 72 or 96 h post infection and euthanized for this purpose at each time point. Blood, liver, kidneys, spleen and small intestines were harvested at the indicated time points and titrated using qRT-PCR. Viral loads were reduced in the kidneys (statistical significance at 72 and 96 h time points with a 12.9- and 5.23-fold decrease) and small intestine (statistical significance at 72 h post infection with a 6.1-fold decrease) in UV-12 treated DENV infected animals as compared those treated with vehicle only (Figure 5) but not reduced in the serum, liver or spleens at the time points sampled in this study. A 5-fold increase in virus titers was observed at the 72 h time point in spleens of the UV-12 treated mice but the groups did not have a difference at the later time point tested.

**Figure 6:**
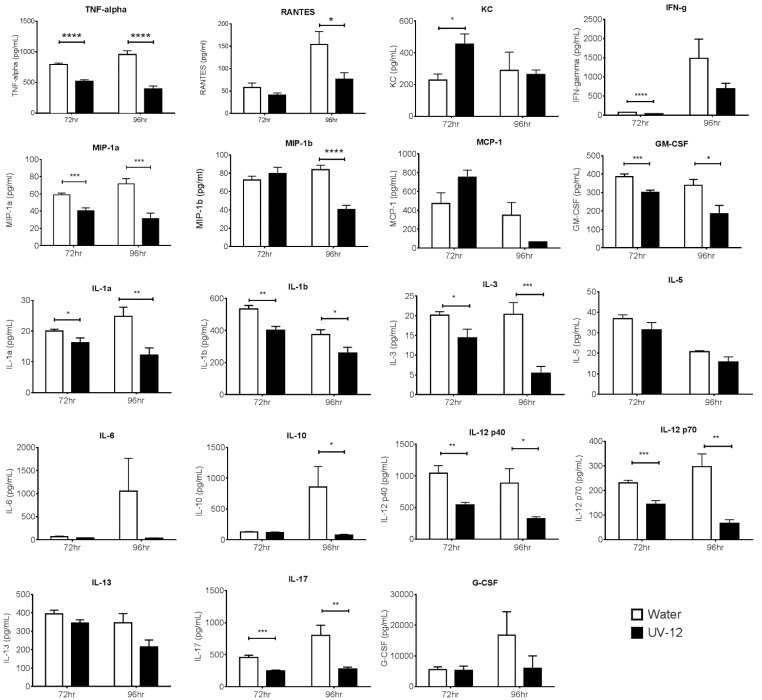
Cytokine levels in serum collected from UV-12 treated AG129 mice. Groups of AG129 mice received the first treatment dose of 100 mg/kg of UV-12 or vehicle control 1 h before infection with DENV2 at a dose of ~1LD_90_. Treatment continued three times daily at 8 h intervals until harvest at 72 or 92 h post infection followed by analysis for levels of G-CSF, GM-CSF, IFN-γ, IL-1α, IL-1β, IL-2, IL-3, IL-4, IL-5, IL-6, IL-10, IL-12 (p40), IL-12 (p70), IL-13, IL-17A, KC, MCP-1 (MCAF), MIP-1α, MIP-1β, RANTES and TNF-α via a multiplex assay. Statistical significance is indicated above sets of data where the *p* value <0.05 (*), <0.01 (**), <0.001 (***), and <0.0001 (****).

Lethal dengue infections, especially in the case of dengue shock syndrome and dengue hemorrhagic fever, are associated with high levels of circulating cytokines and chemokines. Therefore, the effect of UV-12 treatment on cytokine responses was also examined in serum of DENV mice from the samples taken in the study described above. Treatment with UV-12 dampened the overall circulating cytokine levels in the DENV infected AG129 mice as compared to the vehicle only treated mice (Figure 6). Specifically, TNF-α, GM-CSF, MIP-1α, IL-1α, IL-1β, IL-2, IL-3, IL-12p40, IL-12p70, and IL-17 levels were significantly reduced in UV-12 animals compared to vehicle control treated mice at both the 72 and 96 h post infection time points. Levels of IFN-γ were significantly reduced only at the 72 h time point and MIP-1β, RANTES, and IL-10 levels were reduced only at the 96 h post infection time point. There was an apparent reduction in MCP-1 in the UV-12 treated mice at the 72 h time point but the difference was not significant (*p* = 0.0641). KC was the only chemokine that had significantly higher levels (at the 72 h time point only) in UV-12 treated mice as compared to the control animals. Levels of IL-9 and eotaxin were not different (not shown).

### 3.3. Absorption-Distribution-Metabolism-Elimination (ADME), Pharmacokinetic (PK) and Safety Studies

#### 3.3.1. ADME Studies

UV-12 was tested in a standard panel for drug-like properties (*i.e.*, potential for bioavailability, solubility, and metabolic stability) and characterized for safety that predict whether a molecule is likely to be a clinical drug candidate. The findings of the *in vitro* ADMET studies that were conducted for UV-12 are summarized in Table 2.

**Table 2:** *In vitro* ADME properties of UV-12.

**Caco-2 permeability**	Efflux Ratio	4	
**Plasma protein binding (% bound at 10 uM)**	Human	68%	
	Dog	72%	
	Rat	68%	
	Mouse	64%	
**Liver microsome metabolic stability (0.5 mg/mL protein concentration)**		CL_intr_ (mL/min/g liver)	Half-life (min)
	Human	<0.6	>30
	Dog	<0.6	>30
	Rat	<0.6	>30
	Mouse	<0.6	>30
**CYP inhibition (IC_50_)**	CYP1A2	>100 μM	
	CYP2C9	>100 μM	
	CYP2C19	>100 μM	
	CYP2D6	>100 μM	
	CYP3A4^a^	>100 μM	
**hERG Inhibition**		>300 μM	
**Mini-Ames**		Not mutagenic up to 5mg/plate	

As a preliminary assessment of the predicted bioavailability of UV-12, transport across Caco-2 cells was tested as an *in vitro* model of human small intestinal tissues. There is a well-established correlation between the *in vitro* apparent permeability (Papp) across Caco-2 cells and gastric absorption [30]. The efflux ratio (EF) in a Caco2 permeability assay was moderate (EF = 4, Table 2), predicting moderate transport across the gastrointestinal lumen into the blood stream but also indicating it may be a transporter substrate (*ex*. PGP or BCRP).

Another predictor of drug likeness is the degree to which the compound binds to plasma proteins. The more binding to proteins, the less drug that is available in the unbound form to exhibit the desired effect. Plasma protein binding was moderate and comparable across four species ranging from 64% in the mouse to 72% in the dog (Table 2).

More than half of marketed small molecules are cleared by hepatic CYP-mediated metabolism [31]. Liver microsomes are subcellular fractions that contain membrane bound drug metabolizing enzymes including CYP and can be used to predict the metabolism and clearance of a compound *in vivo*. UV-12 was not metabolized by mouse, rat, dog or human liver microsomes (100% parent drug remained after 30 min) predicting good *in vivo* stability and low liability for rapid clearance. The metabolism of drugs can be divided into three stages including modification, conjugation, and excretion. One of the most common metabolic pathways is hydroxylation by cytochrome (CYP) enzymes. Inhibition of the CYP enzymes can increase the duration and intensity of drug exposure. To determine whether UV-12 inhibited any of the most common CYP enzymes, a standard assay format was used. UV-12 did not inhibit the five cytochrome P450s tested (CYP1A2, CYP2C9, CYP2C19, CYP2D6, and CYP3A4 (both midazolam and testosterone substrates)) at concentrations up to 100 μM (Table 2).

The inhibition of the hERG channel is used as a surrogate to predict the potential of cardiovascular risk of a drug candidate. Using a standard patch-clamp assay, concentrations of 0, 30, 100 and 300 μM of UV-12 showed inhibition of hERG current at 6.3%, 18.2%, 4.6% and 19.1% respectively, while the positive control compound propafenone hydrochloride showed 82.9% inhibition. These results suggested that UV-12 is not an inhibitor of the hERG potassium channel (Table 2).

The Ames test is an assay for potential mutagenicity of test articles. Reversion of the strains to enable growth on minimal media is indicative of mutation-inducing nature of the test article. UV-12 was not mutagenic in a mini-Ames assay up to the top dose of 5000 μg/plate (Table 2).

#### 3.3.2. Safety Data for UV-12 in Rodents

To evaluate the toxicity potential and the maximum tolerated dose when administered to guinea pigs, UV-12 was delivered in a single bolus by the intramuscular route at 20, 40, 60, 80 and 100 mg/kg or by IV route at 10, 20, 40, 60 and 80 mg/kg body weight. There were no clinical signs of toxicity or mortality observed in any of the treated dose groups during the observation period of 7 days. There were no gross abnormalities detected at necropsy. Based on these findings, the maximum tolerated dose of UV-12 in guinea pigs by the intramuscular route is >100 mg/kg by the intramuscular route and >80 mg/kg by the IV route, since these were the highest levels tested.

The oral administration of UV-12 to male mice at a dose of 100 mg/kg three times daily for 10 consecutive days resulted in neither mortality nor any noteworthy effects on general health of the animals, body weights, net body weight gains and food consumption. There was a difference, although not statistically significant, in weight of the treated and control animals. At the end of treatment period, treated animals lost an average of 4.53% weight (1.72 g), while control animals gained 3.97% (1.44 g) compared to their starting weight. Amongst the clinical chemistry parameters evaluated, statistically significant increased AST (76%) and decreased ALP (45%) and glucose (29%) in UV-12 treated mice were considered as treatment related. No other parameters were found to be statistically different between the control and treated groups. Upon necropsy, no test-item related gross findings were observed except in the thymus where lymphoid depletion observed microscopically in the UV-12 treated mice was associated with decreased thymus weight.

#### 3.3.3. Pharmacokinetic Study of UV-12 in Rodents

In mice, absorption of an oral pharmacologic UV-12 dose (100 mg/kg) was rapid and demonstrated good oral bioavailability (F) of 77% as shown in Table 3. Plasma clearance (CL) was calculated at 28.22 mL/min/kg following IV administration. Toxicokinetics studies revealed that UV-12 exhibited rapid absorption following dosing with the median time to reach maximum plasma concentration (T_max_, 0.5 h) post dose with a corresponding maximum plasma concentration (C_max_) of 8.88–12.59 µg/mL. Following IV administration of UV-12 solution to mice, the mean plasma clearance (CL) was low (28.22 mL/min/kg, 31% of mouse liver blood flow 90 mL/min/kg). The mean volume of distribution at steady state (Vss) following IV administration was found to be high (1.98 L/kg, 3-fold higher than the total body water of 0.65 L/kg). Following IV administration UV‑12 was eliminated with a mean elimination half-life (T_1/2_) of 1.92 h and AUC_last_ (5.90 μg.h/mL) and AUC_inf_ (5.91 μg.h/mL) were less than after oral administration 43.14 and 45.54 μg.h/mL, respectively.

**Table 3:** Mean pharmacokinetic parameters of UV-12 in male Swiss Albino mice.

Route/Dose(mg/kg)	T_max_ (h)	C_max_ (μg/mL)	AUC_last_^a^ (μg.h/mL)	AUC_inf_^a^ (μg.h/mL)	CL (mL/min/kg)	V_ss_ (L/kg)	T_1/2_ (h)	F ^b^
IV/10	NA	8.88^c^	5.90	5.91	28.22	1.98	1.92	-
PO/100	0.5	12.59	43.14	45.54	NC	NC	NC	77

^a^ AUC_last_ calculated using data through 8 h and AUC_inf_ calculated using data extrapolated to infinity. ^b^ AUC_inf_ and nominal doses were used for bioavailability calculations; ^c^ back extrapolated concentration at time zero; NA: Not applicable; Regression points (4, 8 and 16 h) were selected for both routes to calculate elimination rate constant. NC: Data results were not suitable to calculate Cl, V_ss_, and T_½_ for the oral route.

Mean pharmacokinetic parameters of UV-12 following subcutaneous, intramuscular and IV bolus administration of UV-12 solution in guinea pigs are summarized in Table 4. Following subcutaneous administration of UV-12 solution at a dose of 100 mg/kg body weight to female guinea pigs, the T_max_ was found to be 0.5 h and C_max_ was 11,035 ng/mL. UV-12 was cleared from plasma with apparent half-life (T_1/2_) of 2.21 h. The subcutaneous absolute bioavailability was calculated as 131%. Following intramuscular administration of UV-12 at a dose of 100 mg/kg body weight, the T_max_ was found to be 0.25 h and C_max_ was 11,636 ng/mL. UV-12 was cleared from plasma with a T_1/2_ of 1.99 h. The intramuscular absolute bioavailability was found to be 109%. Following IV bolus administration of UV-12 solution at a dose of 25 mg/kg body weight, the CL of UV-12 was found to be 95.89 mL/min/kg and the Vss was determined to be 5.14 L/kg, which is >8-fold higher than the normal body water of 0.6 L/kg. UV-12 was cleared from plasma with a T_1/2_ of 1.40 h.

**Table 4:** Mean pharmacokinetic parameters of UV-12 in female Hartley guinea pigs.

Route	Dose (mg/kg)	T_max_^a^ (h)	C_max_ (ng/mL)	AUC_last_ (ng.h/mL)	AUC_inf_ (ng.h/mL)	CL (mL/min/kg)	V_ss_ (L/kg)	T_1/2_ (h)	F ^b^
SC	100	0.5	11,035 ± 3165	23,023 ± 5043	23,078 ± 5060	NC	NC	2.21 ± 0.37	131
IM	100	0.25	11,636 ± 2247	19,122 ± 2088	19,168 ± 2094	NC	NC	1.99 ± 0.91	109
IV	25	NA	8855^c^ ± 470	4368 ± 583	4398 ± 599	95.89 ± 12.69	5.14 ± 0.64	1.40 ± 0.09	NA

^a^ T_max_ reported as median (min-max); ^b^ AUC_inf_ and nominal doses were used for absolute bioavailability (F) calculation; ^c^ back extrapolated concentration at time zero; NA: Not applicable for IV route; NC: Data results were not suitable to calculate CL and Vss for the SC and IM routes.

## 4. Discussion

Iminosugars have long been pursued as antivirals and their activity has been previously described *in vitro* (reviewed in [1]) and in animal models of retroviruses [11], herpes simplex virus [10], flaviviruses such as dengue [9,13,14,16] and Japanese encephalitis virus [17] and Ebola virus [15] infections. While several iminosugars have been tested safely in the clinic, when these approved agents were assessed for antiviral activity in humans against viruses such as human immunodeficiency virus [32], hepatitis C virus [8] and dengue [33], only modest reductions in viral titers were observed. We are developing novel iminosugars with enhanced antiviral activity. We have now demonstrated that the novel iminosugar UV-12 has antiviral activity *in vivo* against both INFV and DENV and positive drug-like properties, similar to a related DNJ analog UV-4 [18].

The *in vitro* IC_50_ values against the rat enzymes using natural substrates showing increased inhibitory activity against ER α-glucosidase I compared to ER α-glucosidase II. This opposite situation is seen in the cellular inhibition assay that analyzes the FOS produced by ERAD as biomarkers of α-glucosidase I and II inhibition. In spite of this increased inhibition of α-glucosidase I, in common with many other iminosugars, we have tested the FOS assay shows an initial buildup of monoglucosylated FOS species (α-glucosidase II inhibition) prior to increase in triglucosylated FOS species (α-glucosidase I inhibition) at higher UV-12 concentrations. This apparent discrepancy between isolated enzyme inhibition and FOS analysis can be explained by the kinetics of the reactions. Removal of the first glucose residue by α-glucosidase I and the second glucose residue by α-glucosidase II in cultured cells probably occurs at close to their limiting rates (V_max_), where addition of a competitive inhibitor, such as UV-12, has a limited effect on the observed rate. Removal of the third glucose residue by α-glucosidase II is much slower, suggesting that the rate is not close to V_max_, and under these conditions a UV-12 has a much greater effect on the rate. Since α-glucosidase II and calnexin both compete for the same substrate in the ER (Glc_1_Man_9_GlcNAc_2_-protein), if α-glucosidase II is unable to hydrolyze the substrate bound to calnexin, the presence of calnexin will significantly reduce the free substrate concentration, hence reducing the rate. Alternatively, if α-glucosidase II is able to hydrolyze the substrate bound to calnexin, the presence of calnexin is likely to change the K_m_. An increase in K_m_ would also result in a reduced rate. Both possibilities have the same functional outcome: The rate of removal of the proximal glucose residue is reduced in cells and hence is more sensitive to the presence of a competitive inhibitor, resulting in a greater accumulation of mono-glucosylated glycans as seen in the FOS analysis.

The *in vivo* antiviral activity of ER α-glucosidase inhibitors against INFV had not been demonstrated until our recent report of efficacy of UV-4 against INFV A (H1N1) [18]. Although we were not able to demonstrate inhibition of INFV replication *in vitro* using the yield-TCID assay in MDCK cells, UV-12 efficiently promoted survival in a lethal INFV mouse model. We are currently pursuing *ex vivo* screening models using primary cells and tissues to determine if their use may have a better correlation with *in vivo* efficacy as compared to our use of immortalized cell lines in our work to date. There are a large number of publications that have demonstrated the ability of iminosugars to modulate the glycosylation pattern of the influenza glycoproteins neuraminidase and haemagglutinin [34,35,36,37] and their interactions with calnexin and calreticulin and, in certain cases, reduce virus replication *in vitro* [12]. However, this appears to be a virus strain- and cell type- specific phenomenon that is not fully explained at this time [36,38,39,40,41,42]. Future directions of our work with UV-12 will be to explore activity against a wider panel of INFV (ex. H3N2, H5N1 and H7N9) in addition to a more diverse panel of viruses.

UV-12 inhibited DENV both *in vitro* and in a lethal DENV-2 mouse model. UV-12 promoted survival in the stringent ADE DENV-2 mouse model at both 20 and 100 mg/kg when dosed three times daily when administered starting as late as 48 h after infection. Following treatment with UV-12, reductions in viral loads were only noted in the kidneys and small intestine but not the serum, liver or spleens at the time points sampled in this study. These results are slightly different than the findings for UV-4; however, UV-12 was able to promote survival in the same dose range as UV-4 (10–20 mg/kg) in the DENV-2 ADE mouse model [16]. The relevance of the apparent difference in the ability to reduce the virus load in various tissues when comparing the efficacy of these two molecules is unclear at this time. Treatment of the DENV-2 infected mice with UV-12 resulted in lower levels of circulating cytokines and chemokines including pro-inflammatory molecules such as TNF-α, GM-CSF, MIP-1α, IL-1α, IL-1β, IL-2, IL-3, IL-12p40, IL-12p70, and IL-17 at 72 and 96 h after infection, IFN-γ at the 72 h time point and MIP-1β, RANTES, and IL-10 only at the 96 h time point. The reductions in mortality, disease signs/symptoms, and cytokine levels, are similar to our previous report on related iminosugar, UV-4 [16] that is currently being evaluated in a Phase 1 clinical trial. The reason for the overall decreased cytokine response observed in UV-12 treated mice, as compared to the vehicle control mice, is not clear. It could either be a result of decreased viral loads, symptomology and subsequent immune responses, changes in the morphology or structure of dengue virus produced in the presence of UV-12 that stimulate inflammatory responses [43] or be linked to direct effects on cytokine production mediated directly by UV-12. The mechanism for cytokine modulation by UV-12 in DENV infections will need to be further explored.

In general, the iminosugar class has demonstrated good drug-like ADMET (absorption, distribution metabolism, elimination, toxicology) characteristics. Molecular weights, logP, and total polar surface area are generally low, which imply good solubility. Indeed, UV-12 was soluble at more than 100 mg/mL in acidified water, which was the highest concentration tested. UV-12 was not metabolized by liver microsomes nor did it inhibit five of the most common CYP isoenzymes. UV-12 had moderate plasma protein binding properties, was readily detected in mouse and guinea pigs at high concentrations after oral administration, and was orally bioavailable as had been predicted by the Caco-2 cell permeability test. The PK of UV-12 was similar in mice and guinea pigs. Specifically, the T_max_ was 0.25–0.5 h, the C_max_ was 8.85–11.64 µg/mL, and the AUC and T_1/2_ for mice and guinea pigs were similar following UV-12 dosing. UV-12 was well tolerated in a repeat dose study for 10 days at 100 mg/kg. All these tests point to good drug-like properties for UV-12 but further work in additional species would be required should this molecule be selected for future development.

There are currently three iminosugar compounds in clinical use: Miglustat (Zavesca^®^) for the treatment of Gaucher’s disease and Niemann-Pick type C, and miglitol (Glyset^®^) and acarbose (Precose^®^) for the treatment of type II diabetes mellitus. In addition, two iminosugars are currently in development for the treatment of DENV including celgosivir (a pro-drug of castanospermine) that has been tested in several clinical studies and a hydrochloride salt of UV-4 that is currently being tested in a Phase 1 clinical trial. Together, these compounds provide a large safety database for the evaluation of the iminosugar class of compounds. The recent publication by Sadat *et al.* described two siblings with CDG-IIb having multiple neurologic complications and severe hypogammaglobulinemia, which may be associated with the absence of ER α-glucosidase I. However, it should be noted that these children have complete and permanent absence of ER α-glucosidase I including during fetal development. Notably, the approved status of several other iminosugars for chronic use provides strong evidence that iminosugars related to DNJ present no significant toxicological concerns. Partial blockage of host ER α-glucosidases is not grossly deleterious to the host, as evidenced by long-term usage of NB-DNJ for treatment of Gaucher’s disease. The proposed use of optimized iminosugar candidate(s) as an antiviral for acute viral diseases is not likely to be more than 7 to 10 days dosage, thus limiting the duration of inhibition of the host enzymatic pathways and reducing risk for mechanism-related side effects. Encouragingly, when mice were treated with 100 mg/kg/dose (300 mg/kg/day) of UV-12, the only remarkable finding was lymphoid depletion in the thymus being observed microscopically in the UV-12 treated mice and associated with decreased thymus weight. Thymic acellularity has been noted before when DNJ-based iminosugars have been administered to the mouse. NB-DNJ administration leads to thymus weight loss but the effects are rapidly reversed following drug removal and seem to have no functional, *i.e.*, immunological, consequence [20,44].

Iminosugars are glucomimetics with positive drug like properties that have been well known for many years and several are approved for use in humans. The iminosugars are ER α-glucosidase inhibitors that have antiviral activity with a reduced likelihood for generation of antiviral resistance due to targeting of host-based mechanisms. For these reasons, iminosugars offer an attractive avenue for developing broad spectrum antiviral therapies. Iminosugars with significant potency and good drug-like properties should be pursued as clinical candidates for treatment of diverse viral infections. UV-12 or more potent derivatives will be further studied and considered for advanced development.
